# Fatigue resistance of rat extraocular muscles does not depend on creatine kinase activity

**DOI:** 10.1186/1472-6793-5-12

**Published:** 2005-08-17

**Authors:** Colleen A McMullen, Katrin Hayeß, Francisco H Andrade

**Affiliations:** 1 Department of Physiology, University of Kentucky, 800 Rose St., Lexington KY 40536-0298, USA; 2 Department of Cell Biology, University of Potsdam, D-144171 Potsdam, Germany

## Abstract

**Background:**

Creatine kinase (CK) links phosphocreatine, an energy storage system, to cellular ATPases. CK activity serves as a temporal and spatial buffer for ATP content, particularly in fast-twitch skeletal muscles. The extraocular muscles are notoriously fast and active, suggesting the need for efficient ATP buffering. This study tested the hypotheses that (1) CK isoform expression and activity in rat extraocular muscles would be higher, and (2) the resistance of these muscles to fatigue would depend on CK activity.

**Results:**

We found that mRNA and protein levels for cytosolic and mitochondrial CK isoforms were lower in the extraocular muscles than in extensor digitorum longus (EDL). Total CK activity was correspondingly decreased in the extraocular muscles. Moreover, cytoskeletal components of the sarcomeric M line, where a fraction of CK activity is found, were downregulated in the extraocular muscles as was shown by immunocytochemistry and western blotting. CK inhibition significantly accelerated the development of fatigue in EDL muscle bundles, but had no major effect on the extraocular muscles. Searching for alternative ATP buffers that could compensate for the relative lack of CK in extraocular muscles, we determined that mRNAs for two adenylate kinase (AK) isoforms were expressed at higher levels in these muscles. Total AK activity was similar in EDL and extraocular muscles.

**Conclusion:**

These data indicate that the characteristic fatigue resistance of the extraocular muscles does not depend on CK activity.

## Background

In order to maintain a high effective free energy change of ATP hydrolysis (ΔG_ATP_), metabolically active tissues need to control cellular ATP and ADP levels within a relatively narrow range. This is particularly true in skeletal muscle, as its energy requirements can fluctuate rapidly by more than two orders of magnitude. In mammalian skeletal muscles and other tissues with widely fluctuating metabolic needs, the creatine-phosphocreatine system buffers intracellular ATP concentration: creatine kinase catalyzes the reversible transfer of the phosphoryl group from phosphocreatine to ADP and sustains normal ATP levels [1]. Cellular creatine kinase (CK) activity is due to a family of oligomeric enzymes: two cytosolic, ubiquitous "brain-type" CK-B and "muscle-type" CK-M, and two mitochondrial isoforms, ubiquitous mitochondrial CK (uCK) and "sarcomeric" mitochondrial CK (sCK). The mitochondrial CK isoforms are found as homo-octamers in the inner mitochondrial membrane [2,3]. The cytosolic -M and -B subunits form homo- and heterodimers, CK-MM, -MB- and -BB isoenzymes [2]. In differentiated skeletal muscle, CK-MM and sCK are the predominant isoforms. In fast-twitch muscles, most CK activity is due to the CK-MM isoform, which is preferentially associated with the sarcomeric M line, the sarcoplasmic reticulum and T-tubules [1,4]. This arrangement couples the CK-dependent ATP buffering system to the cellular sites with the highest ATPase activity, and is important for normal contractile function [5,6]. In addition, targeting of CK to these cellular microenvironments localizes CK activity where it is needed, optimizing enzyme distribution [7].

The extraocular muscles, responsible for voluntary and reflexive movements of the eyes, are arguably the fastest and most active skeletal muscles [8-10]. These muscles are typically activated in a biphasic fashion: a high-intensity burst followed by a lower-frequency step (pulse-step) [11]. These functional properties depend on a reliable energy supply and suggest that the extraocular muscles may rely on cytosolic CK-M activity to a great extent. Furthermore, the extraocular muscles are characterized by abundant mitochondria and the presence of developmental and cardiac markers. Then, this muscle group may potentially express CK-B and mitochondrial CK isoforms at higher levels than limb skeletal muscles. Therefore, we tested the hypotheses that (1) the expression and content of CK isoforms and CK activity in rat extraocular muscles would be higher than in fast limb skeletal muscle, and (2) that the fatigue resistance of the extraocular muscles would be critically dependent on normal CK activity. The results showed that the expression and content of muscle-specific cytosolic and mitochondrial CK isoforms is actually lower in extraocular muscle than in extensor digitorum longus (EDL), a prototypical fast limb skeletal muscle. Hence, total CK activity in extraocular muscle is significantly less than in EDL. In addition, the fatigue resistance of the extraocular muscles is not altered when CK activity is inhibited. These data are further evidence that the extraocular muscles manage the metabolic load imposed by their constant activity in a manner not usually seen in typical fast skeletal muscles.

## Results

### Lower CK activity and isoform expression in extraocular muscle

**Figure 1:**
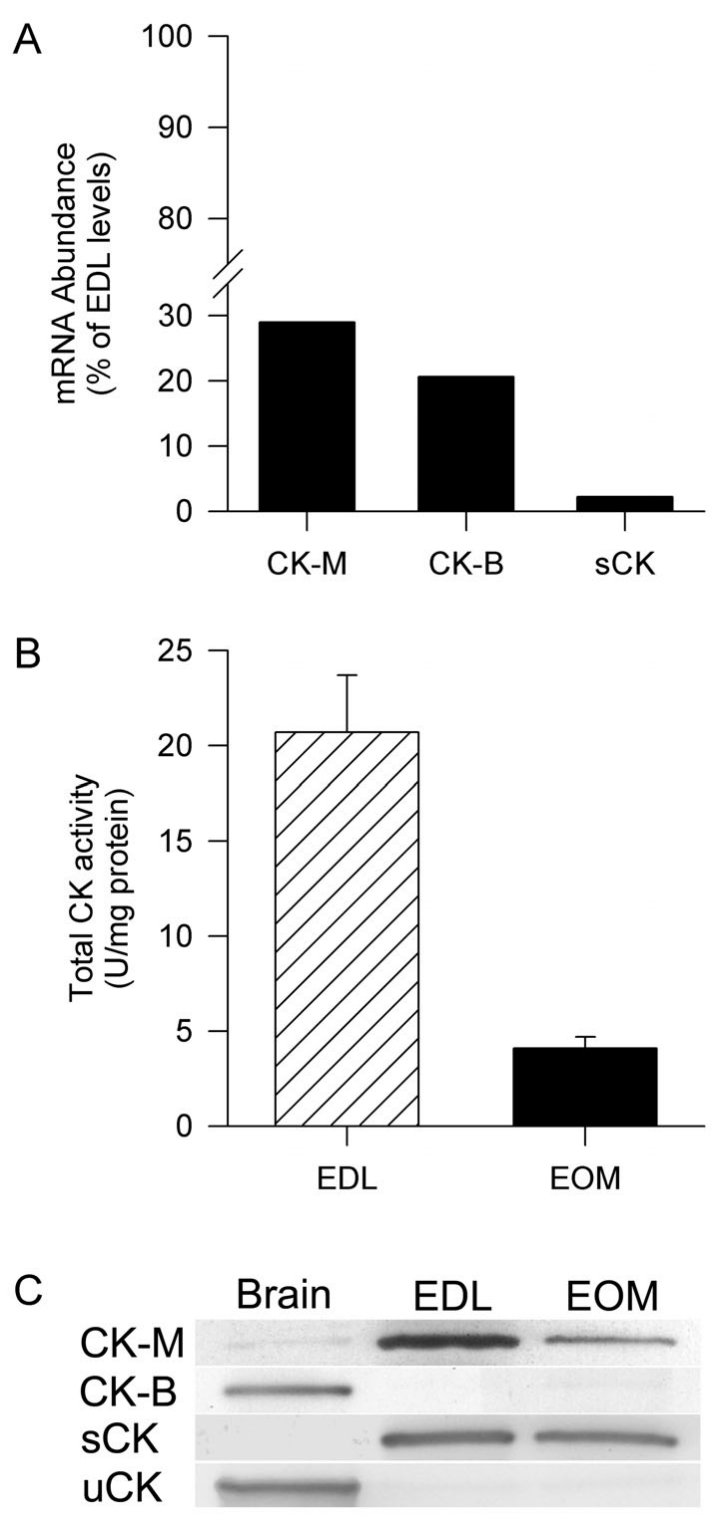
**Lower CK isoform expression and activity in rat extraocular muscle**. (A) Relative abundance of three CK isoform mRNAs in EDL and extraocular muscle by quantitative PCR. Each sample includes pooled muscles from 4 rats. Data are means from 3–4 samples, and are normalized to levels measured in EDL = 100%. Message levels for the cytosolic CK-M and CK-B, and the mitochondrial sCK were much lower in extraocular muscles compared to EDL. (B) Total CK activity in EDL and extraocular muscles. EDL muscles have ~5-fold greater CK activity than extraocular muscles (n = 8, P < 0.001). (C) CK protein isoforms found in brain, EDL and extraocular muscle by western blot.

We used quantitative PCR to compare the expression of all the CK isoforms in the extraocular muscles and EDL. Message for the main cytosolic CK isoform in skeletal muscle, CK-M, was decreased in the extraocular muscles, as was the other cytosolic isoform, CK-B (figure 1A). The lower expression of cytosolic CK isoforms in extraocular muscle does not result in a compensatory increase in the expression of the mitochondrial CKs: mRNA abundance for muscle-specific sCK was also significantly less in the extraocular muscles (figure 1A); uCK was found in EDL by quantitative PCR, but it was below detectable levels in the extraocular muscles. It can then be inferred that uCK expression in these muscles is also significantly lower than in EDL, although no relative comparison could be calculated. In turn, total CK activity in rat extraocular muscles was only ~20% of that in the fast-twitch muscle EDL (4.1 ± 0.6 *vs. *20.7 ± 3.0 U/mg protein, respectively, figure 1B). Figure 1C shows western blots that demonstrate that CK-B and uCK isoforms were not detectable in EDL and extraocular muscles. The muscle-specific isoforms CK-M and sCK were found in the two muscles but not in brain. CK-M protein content was less in the extraocular muscles. Although the mitochondrial volume density of the extraocular muscles is ~3- to 4-fold higher than in EDL (McMullen and Andrade, not shown), sCK protein was also less abundant in the extraocular muscles.

### Less CK binding sites in extraocular muscle

**Figure 2:**
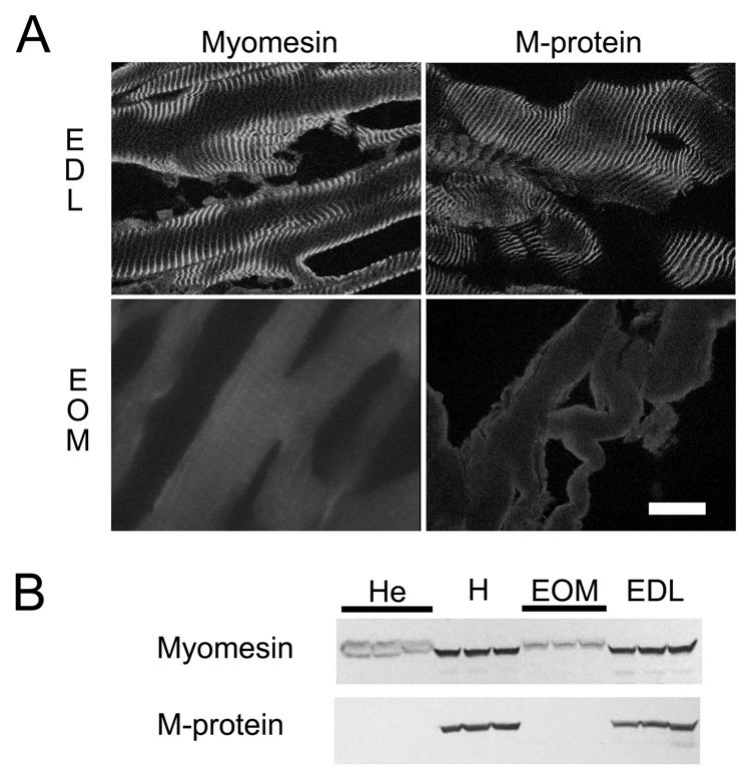
**Myomesin and M-protein are downregulated in rat extraocular muscles**. (A) Representative confocal micrographs showing that myomesin and M-protein are found in EDL muscle fibers (EDL, top) in the same stereotypical banding pattern, but are not detectable in extraocular muscle (EOM, bottom). Scale bar = 20 μm. (B) Western blots demonstrating the presence of myomesin and M-protein in EDL and heart (H). Embryonic heart (He) did not have M-protein, but two bands reacted with the myomesin antibody: myomesin and EH-myomesin, an alternatively spliced isoform of higher molecular weight. Extraocular muscle (EOM) contained EH-myomesin, but did not have detectable levels of myomesin or M-protein.

A fraction of CK-M in skeletal muscles associates with the sarcomeric M lines. Rat extraocular muscle fibers do not have M lines [12,13]. Myomesin and M-protein, the main cytoskeletal components of the M lines, were not detected in extraocular muscles by immunocytochemistry, but were clearly found in EDL fibers in a stereotypical banding pattern (figure 2A). Western blotting failed to detect M-protein in the extraocular muscles (figure 2B). On the other hand, the extraocular muscles contained an alternatively spliced isoform of myomesin, EH-myomesin. This isoform was also detected in embryonic heart together with myomesin (figure 2B). The level of M-protein mRNA measured with quantitative PCR was correspondingly lower in extraocular muscle (13.9-fold less than in EDL); however, myomesin mRNA content was higher (2.1-fold higher than in EDL).

### Effect of CK inhibition on fatigue development

The baseline *in vitro *contractile properties of extraocular and EDL muscles are presented in Table 1. Twitch kinetics (time to peak twitch force, half-relaxation time, and twitch-to-tetanus ratio) were significantly lower in the extraocular muscles than in the EDL muscle bundles. Maximal tetanic forces (P_0_) of the extraocular muscles were only ~25% of the P_0 _generated by EDL bundles. Although the contractile properties of the extraocular muscles are very different from those of EDL, they are similar to those reported previously by us and others [14,15].

**Figure 3:**
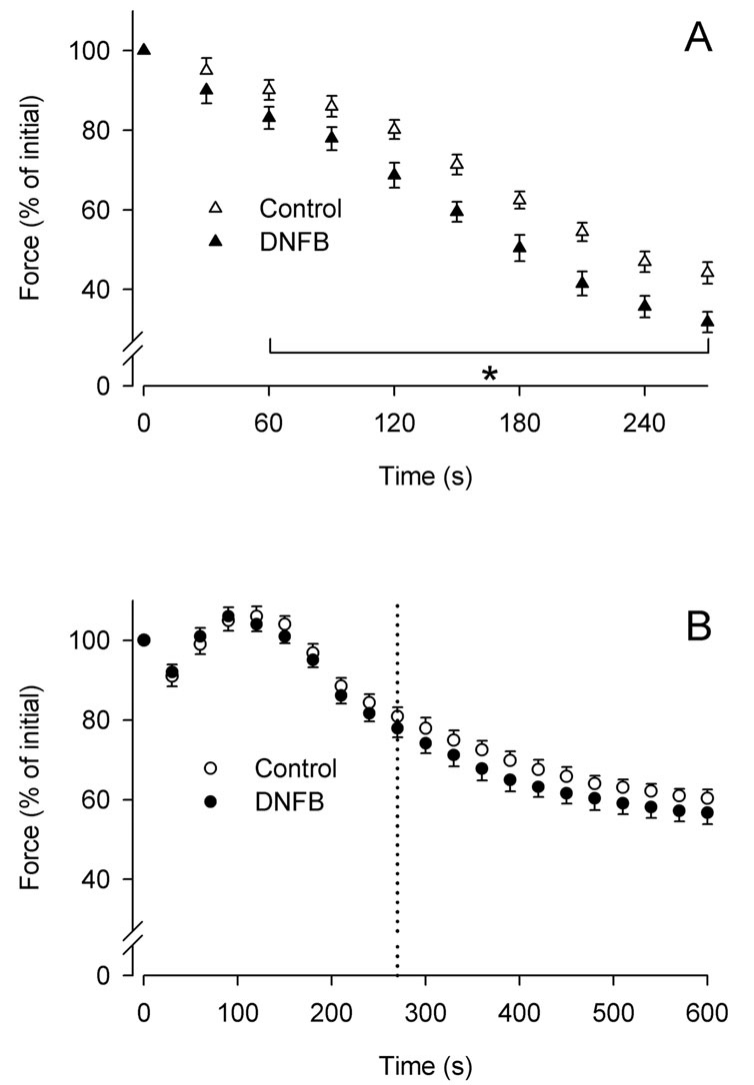
**CK inhibition does not alter fatigue resistance of rat extraocular muscle**. (A) DNFB (black triangles) decreases the fatigue resistance of EDL muscle bundles (n = 6 muscles per treatment, * P < 0.05 control *vs. *DNFB at the corresponding time points). (B) DNFB (black circles) does not accelerate fatigue development in extraocular muscles (n = 6 muscles per treatment). Vertical dotted line is included for comparison to mark to the end of the fatigue protocol for EDL muscles.

In our initial experiments, extraocular muscles and EDL bundles were incubated with the CK inhibitor 2,4-dinitro-1-fluorobenzene (DNFB) immediately following the determination of baseline contractile properties. To check for non-specific DNFB-induced toxicity, its effect on P_0 _was measured with maximal tetanic contractions at 2-min intervals for up to 30 min. DNFB at 50 μM resulted in a drastic loss of force in EDL bundles after only 6 min; P_0 _decreased by 22.4 ± 3.5% from pre-DNFB baseline (n = 4 muscles, P < 0.05). The magnitude of the force drop in extraocular muscles after 6 min in 50 μM DNFB was less but still significant: P_0 _decreased by 12.1 ± 2.1% (n = 4 muscles, P < 0.05). On the other hand, DNFB at 10 μM for = 20 min did not alter force significantly in either muscle group: EDL 98.6 ± 1.1% of baseline, and extraocular muscles 97.7 ± 2.0%, n = 4 muscles/each, P = not significant. Therefore, for the rest of the study we incubated muscles in 10 μM DNFB for 20 min, and then washed it out for 10 min before the start of the fatigue protocol. Five minutes before the start of fatigue, during the washout period, P_0 _was measured to ensure the functional integrity of the muscles. Fatigue was induced with 500-ms submaximal tetani (~50% of peak tetanic force) at 1.5 s intervals until force declined by 50% or for 10 min, whichever occurred first. Untreated control extraocular muscles sustained forces above 50% of initial for the full duration of the fatigue protocol. Control EDL bundles, on the other hand, sustained forces above 50% of the initial for only 4.8 ± 0.5 min. DNFB accelerated the development of fatigue in EDL bundles, such that 60 s into the fatigue protocol there was already a significant difference between the control and DNFB-treated muscles (figure 3A). At the end of the fatigue protocol (270 s), control EDL muscles generated 44 ± 3% of the initial force *vs. *32 ± 3% for DNFB-treated EDL bundles (P < 0.05). By contrast, the extraocular muscles proved more resistant to DNFB; the changes in force during the fatigue protocol followed almost the same trajectory with and without DNFB, and after 10 min peak forces were not significantly different: control 60 ± 2 and DNFB 57 ± 3% of initial force (figure 3B).

### Upregulation of AK isoforms in extraocular muscle

**Figure 4:**
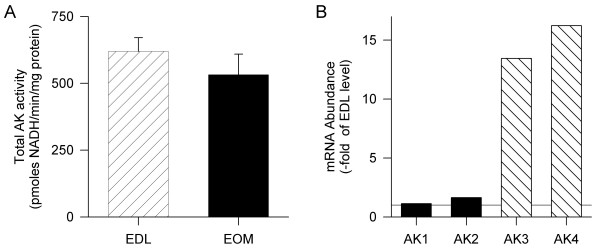
**Upregulation of AK isoforms in rat extraocular muscle**. (A) Total AK activity in EDL and extraocular muscles (n = 9, P > 0.35). (B) Relative abundance of mRNAs for four AK isoforms in extraocular muscle by quantitative PCR. Each sample includes pooled muscles from 4 rats. Data are mean -fold differences (3–4 samples) in AK isoform expression and normalized to EDL muscle = 1.0, shown by the horizontal reference line. The abundance of AK1 and AK2 mRNAs was not significantly different in extraocular and EDL muscles (black bars). AK3 and AK4 are expressed at significantly higher levels in extraocular muscle (13.5- and 16.2-fold greater than EDL, respectively).

The "myokinase" reaction (2 ADP → ATP + AMP), catalyzed by AK, serves as an alternative ATP buffering system in skeletal muscle. Total AK activity in extraocular muscles was ~86% of the activity measured in EDL muscles, and this difference was not statistically significant (figure 4A). There are four known AK isoforms in rats, two of which (AK1 and AK2) are found at relatively high levels in skeletal muscle [16,17]. We used quantitative PCR to determine the relative expression of the different AK isoforms in EDL and extraocular muscles. AK1 and AK2 were detected at equivalent levels in the extraocular and EDL muscles; differences were less than the 2-fold threshold for significance (Figure 4B). However, mRNAs for AK3 and AK4 were over 13-fold more abundant in the extraocular muscles.

## Discussion

The results from this study demonstrate that CK expression, content and activity are significantly lower in rat extraocular muscles and that CK activity does not explain the fatigue resistance of this muscle group, leading us to reject our initial hypotheses.

### Decreased CK expression and activity in extraocular muscle

CK is particularly abundant in fast-twitch skeletal muscles; therefore, we measured total CK activity and CK isoform expression in EDL, a prototypical fast limb muscle, and in the extraocular muscles, which are also composed mostly of fast-twitch fibers. Surprisingly, CK activity was significantly less in the extraocular muscles, only 20% of the level measured in EDL. Message abundance and protein content of the muscle-specific CK-M were also lower in the extraocular muscles (figure 1). A fraction of CK-M is normally associated with the sarcomeric M lines, structures that support the thick filament lattice and are more prominent in fast-twitch muscle fibers [4,6]. In consequence, we explored whether low CK-M content correlated with the absence of M lines characteristic of most rat extraocular muscle fibers [12,13]. Our results confirmed the previously described altered expression pattern of the M line cytoskeletal components, myomesin and M-protein, in rodent extraocular muscles (figure 2) [13,18]. The lack of M-protein is particularly intriguing since this cytoskeletal protein is found almost exclusively in fast twitch fibers [19,20]. This finding also correlates with the description of fainter M lines in skeletal muscles from CK-M knockout mice [21]; apparently the association of the enzyme with the M lines contributes importantly to the electron density of these structures. Myomesin was also less abundant in extraocular muscle, as shown by immunocytochemistry and western blot, despite the fact that message levels were higher than in EDL. This finding is at variance from our recent study of mouse extraocular muscles [18]. It may indicate that post-transcriptional control of myomesin expression is relatively more important in the extraocular muscles of adult rats. Western blotting confirmed that rat extraocular muscles contain an alternatively spliced myomesin isoform, EH-myomesin, originally described in embryonic heart [22,23]. The fact that this isoform was not detected by immunocytochemistry in the present study may reflect low protein abundance.

Extraocular muscles express developmental and cardiac markers [24]. Since the CK-MB heterodimer is the prevalent CK isoform in developing skeletal muscles and in adult myocardium, we anticipated a relative upregulation of CK-B in extraocular muscle compared to EDL. Our results did not bear this out; CK-B mRNA and protein were also significantly less in the extraocular muscles (figure 1).

Interestingly, the fast-twitch skeletal muscle fibers from CK-M knockout mice have higher mitochondrial contents and increased sCK activity [21]. Extraocular muscle fibers have very high mitochondrial contents [12]. It has been suggested that a greater number of mitochondria reduces the diffusion distance between these organelles and myofibrills and other ATP sinks, compensating for the loss of cytosolic CK [21,25]. By analogy, the high mitochondrial content of extraocular muscles would achieve the same goal, minimizing the need for the temporal and spatial ATP buffering capacity normally provided by cytosolic CK activity. Then, the movement of ATP from mitochondria to cytosolic phosphocreatine may not be required in the extraocular muscles. Despite the high mitochondrial content of extraocular muscles, mRNA and protein levels of sCK and uCK were lower in these muscles; uCK was actually undetectable in the extraocular muscles by quantitative PCR. The lower expression and content of sCK are particularly puzzling; it has been proposed that sCK mediates the high-energy phosphoryl flux from mitochondria to cytosol and regulates oxidative phosphorylation [26].

### CK inhibition and fatigue resistance

The most physiologically significant finding in this study was that CK inhibition with DNFB did not change the fatigue resistance of the extraocular muscles, even though it significantly accelerated the development of fatigue in the fast-twitch muscle EDL (figure 3). The lack of effect of DNFB on extraocular muscle fatigue was more impressive because these muscles were stimulated at a higher frequency than the EDL bundles in order to fulfill the requirement that all muscles start the fatigue protocol at 50% of maximal force. In addition, endurance (time to decrease force by 50%) was much longer in the extraocular muscles, since all EDL bundles reached this target force by 3 min, and all extraocular muscles sustained forces >50% of initial for 10 min. The use of phosphocreatine in skeletal muscle energetics allows for high power output and effective buffering of ATP concentration. While maintenance of constant ATP concentration during contractile activity is neither universal not absolutely required, the use of phosphocreatine as fuel increases the power output attainable by skeletal muscles. This and delaying fatigue after the onset of high-intensity stimulation are the most perturbed functions in CK knockout mice [21,25]. However, CK activity may actually contribute to fatigue development during prolonged stimulation by increasing the cytosolic concentration of inorganic phosphate [27]. CK would then be deleterious to a muscle group that is constantly active and whose energy requirements may not rely on traditional ATP buffers [28,29]. Because most skeletal muscle fibers are recruited during fairly short periods, peak energy demand is much greater than the average demand [30]. During these sporadic periods of peak demand, CK provides the "metabolic capacitance" needed to allow the system to have only enough mitochondria to provide for average energy demand. Such arrangement may not serve constantly active muscles such at the extraocular muscles. Interestingly, skeletal muscles with genetically impaired CK activity resemble the normal extraocular muscle phenotype: high mitochondrial content, low force and power [12,31]. Both phenotypes increase ATP generating capacity and decrease the size of the ATP sinks.

### Upregulation of AK isoforms in extraocular muscle

Preferential targeting of CK to myofibrillar and mitochondrial compartments serves to localize activity to where it is needed, thus economizing enzyme distribution. This would be particularly important in typical skeletal muscle fibers where sparsely and non-uniformly distributed mitochondria impose large diffusion distances [7]. But it would be much less so in the mitochondria-rich and very small extraocular muscle fibers, explaining the lack of M lines and low CK activity. Instead, mRNAs for the mitochondria-associated AK3 and AK4 isoforms are present at higher levels in these muscles, confirming an earlier gene expression profile study that found AK4 at higher levels in mouse extraocular muscles [28]. Interestingly, AK activity is increased in CK-deficient mice, and inhibition of CK in intact skeletal muscle results in increased phosphoryl transfer via AK [32,33]. However, total AK activity was not significantly different between EDL and extraocular muscle. Whether the putative mitochondrial localization of AK3 and AK4 is designed to replace CK activity associated with mitochondria in the extraocular muscles remains to be determined.

Another possible role for AK in extraocular muscle is that it may mediate an alternative strategy for metabolic control. The lower expression of mitochondrial CK in extraocular muscles would diminish its proposed role for fine regulation and amplification of the energy state signal from the cytoplasm and control of mitochondrial oxidative phosphorylation [26,34]. Instead, AMP from the AK-catalyzed myokinase reaction would serve as a strong positive allosteric signal on 6-phosphofructo-1-kinase (PFK) and inhibit fructose 1,6-bisphosphatase, activating glycolysis. This control step could be particularly important in the extraocular muscles given their apparent reliance on fatty acid oxidation and glycolysis and not on glycogen breakdown [28,29].

## Conclusion

Our data indicate that the fatigue resistance of the fast and constantly active extraocular muscles does not depend on high CK activity. In consequence, the expression of the known CK isoforms is downregulated in these muscles. These findings strengthen emerging evidence that the extraocular muscles follow a different strategy or design to cope with their peculiar mechanical and metabolic loads.

## Methods

### Animals

Ethical use of experimental animals was approved by the Institutional Animal Care and Use Committee. Three-month old male Sprague Dawley rats (Harlan, Indianapolis, IN) were anesthetized with ketamine hydrochloride/xylazine hydrochloride (100 mg/8 mg per kg body weight, i.p. injection) and killed by exsanguination following a medial thoracotomy. For *in vitro *function, extraocular muscles were dissected intact from bony origin to distal tendon. Small EDL bundles were taken to yield a representative fast limb skeletal muscle sample. For gene expression and protein studies, all extraocular muscles and mid-belly samples of EDL were quickly excised, frozen in liquid nitrogen and stored at -80°C. For immunocytochemistry, whole muscles were dissected, pinned to cork at resting length, covered with OCT embedding medium and frozen in 2-methylbutane cooled in liquid nitrogen.

### Enzyme assays

EDL and extraocular muscle samples were homogenized (1:100 w/v) in 26 mM Tris, 30 mM dithiothreitol, 0.3 M sucrose and 1% Triton X-100 (pH 8.0), and extracted on ice for 1 hr. CK activity was determined in triplicate by a hexokinase/glucose-6-phosphate dehydrogenase coupled system, which yields NADH at a rate proportional to CK activity (Sigma Chemical Co., St. Louis, MO), and expressed as units/mg of protein. AK activity was determined in triplicate by a pyruvate kinase/lactate dehydrogenase coupled system which follows the consumption of NADH, and expressed as pmoles NADH/min/mg of protein. Protein content of muscle homogenates was determined by the Lowry method [35].

### Real-time quantitative PCR

Muscles were pulverized under liquid nitrogen and total RNA was isolated using Trizol (GibcoBRL, Rockville, MD) according to the manufacturer's instructions. Muscles from four animals were combined into each total RNA sample to lessen the effect of inter-subject variability. Reverse transcription was carried out using Superscript II RNAse H^- ^Reverse Transcriptase (Invitrogen, Carlsbad, CA) with random hexamers. Primers for the mRNAs of interest were designed with the software package Primer Express version 1.5 (Applied Biosystems, Foster City, CA) from GenBank nucleotide sequences and are shown in Table 2. cDNA samples (2 μg each) were analyzed in triplicate with the ABI Prism 7700 Sequence Detection System (Applied Biosystems) using ABI SYBR Green and β-actin as the calibrator housekeeping gene. The relative abundance of target mRNAs in the extraocular and EDL muscles was determined with the comparative cycle threshold method [36,37].

### Immunocytochemistry

Five-μm thick longitudinal cryosections from extraocular and EDL muscles were fixed with 2% paraformaldehyde, blocked with 0.1% bovine serum albumin in phosphate-buffered saline, and incubated overnight at 4°C with monoclonal antibodies specific for myomesin or M-protein (BB78 and AA259, respectively) [38]. After washing with phosphate-buffered saline, slides were incubated for 1 h in Alexa Fluor 488-conjugated secondary antibody (1:50; Molecular Probes Inc., Eugene, OR), rinsed in phosphate-buffered saline, and mounted in Immu-Mount (Shandon, Pittsburgh, PA). Sections were examined and imaged with a Zeiss LSM 410 confocal microscopy system.

### Immunoblotting

For the analysis of myomesin, EH-myomesin, M-line protein and CK isoform content, tissue samples were homogenized in a buffer containing 26 mM Tris-HCl, 0.3 M sucrose, 30 mM DTT and 1% Triton X-100; pH was adjusted to 8.0. Western blots for myomesin, EH-myomesin and M-protein were carried out using previously described monoclonal antibodies and chemiluminescence [38]. Polyclonal antisera to synthetic peptides identical to rat amino acid sequences of the cytosolic CK-M and CK-B (Rockland Immunochemicals), and sCK and uCK (Affinity Bioreagents) were raised in New Zeland white rabbits and collected by terminal bleeding. Specificity of antisera was determined by ELISA and dot blot. Western blots for CK isoforms were carried out as follows: 50 μg of total protein per sample were electrophoresed and transferred to PVDF membranes. Blocked membranes were probed with CK isoform-specific sera (1:1000), followed by a horseradish peroxidase-conjugated secondary antibody (Bio-Rad). Membranes were then developed with 4-chloro-1 naphthol, scanned and analyzed using ImageJ 1.30v [39].

### Isolated muscle preparation

Whole extraocular muscles and small EDL muscle bundles (~10–15% of total muscle mass) were placed in a small muscle chamber. The distal tendon was attached to a micropositioner and the proximal bone fragment to a force transducer (AE801, SensoNor, Horten, Norway or ELG-H, Entran, Fairfield, NJ). The chamber was superfused with a physiological salt solution: (in mM) 137 NaCl, 5 KCl, 2.0 CaCl_2_, 1.0 MgSO_4_, 1.0 Na_2_HPO_4_, 24 NaHCO_3_, 11 glucose, and 0.026 d-tubocurarine, bubbled with a 95% O_2_-5% CO_2 _gas mixture to maintain pH at 7.4 at 25°C. To inhibit muscle CK activity, 2,4-dinitro-1-fluorobenzene (DNFB, Sigma Chemical Co.) was added to the bath from a 100 mM stock solution in dimethyl sulfoxide (DMSO) [33,40,41]. Muscles were stimulated with 0.5 ms pulses delivered by an S48 stimulator (Grass Instruments, Braintree, MA) to platinum electrodes in the muscle chamber and stretched to the length giving maximum tetanic force (optimal length, L_0_). Force signals were stored in a personal computer for analysis. At the end of the study, the length of muscle fibers at L_0 _was measured and bone and tendons removed. The muscles were blotted dry and weighed. Force (in Newtons) was normalized to muscle cross sectional area (cm^2^) [42].

### Fatigue protocol

After measuring baseline contractile properties, muscles were divided into two groups: DNFB-treated and time-matched controls. Pilot studies demonstrated that 30-min exposures to DMSO at the concentrations used here (0.01 to 0.05%) followed by 10-min washout had no effect on the response to the fatigue protocol. Fatigue was induced with the following protocol: muscles were stimulated at a frequency giving approximately one half of maximal tetanic force (30–50 Hz for EDL bundles, 50–70 Hz for extraocular muscles) for 500 ms, followed by 1.5 s interval between contractions, until force in the control group declined to approximately 50% of the level at time = 0 or for 10 min, whichever occurred first.

### Data analysis

All results are presented as means ± s.e.m. of *n *observations, unless otherwise noted. Statistical significance was determined at the 95% confidence level using Student's *t *test for unpaired or paired samples as indicated; the treatment effect in the fatigue runs was determined by analysis of variance.

## Authors' contributions

CAM was responsible for real-time quantitative PCR, immunocytochemistry and confocal microscopy. KH contributed the immunoblotting of M line proteins. FHA performed the functional studies, enzyme assays and immunoblotting of CK isoforms. All authors participated in the experimental design and manuscript preparation.

## Figures and Tables

**Table 1:** Contractile properties of EDL and extraocular muscles

	TPT (ms)	HRT (ms)	P_T_/P_0 _(%)	P_0 _(N/cm^2^)	*n*
EDL	25.9 ± 0.5	25.4 ± 0.5	24.6 ± 1.7	24.2 ± 0.4	16
EOM	13.2 ± 0.4*	13.4 ± 0.4*	8.0 ± 0.6*	6.4 ± 0.2*	16

TPT, time to peak twitch force; HRT, half-relaxation time; P_T_/P_0_, twitch-to-tetanus ratio; P_0_, maximal tetanic force. EOM = extraocular muscles. * P < 0.001 EDL *vs. *extraocular muscle.

**Table 2:** Primer sequences for quantitative PCR

Name	Size (bp)	Forward / Reverse primers (5' → 3')
CK-M	76	GCCATGGTGGCTTCAAACC / CAGATCGTCTCCACCCTTGAG
CK-B	67	AAGCCCTGTCCAGCCTAGATG / CGCCTCGGTCATGCTCTT
sCK	73	GAGCTGGTGTCCACGTTAGGA / CCGCAGGTTCTCCAAGATCTT
uCK	82	CTGACCTTGATGCCAGCAAA / ACTTCGGCCAGTTCTGACTCTT
AK1	78	CTTCCAACGGCTTCTTGATTG / TGTGCGATCTTCCGTTCAAA
AK2	71	TGGAGGCCTACCACATCAGA / CAATGGCGCAGTGAATGA
AK3	75	ACCAGTTGTAGCTGGCTGTTG / TCTGATAAACTCTCCAGGGCTTCT
AK4	71	CTTCCAGCGGGAGGGTCTTAT / CGCCGGTGATGTCATCAAC
Myomesin	75	GGCCCACATTTCGCTGAGTA / TTGCCACCTTGCATTTCAAC
M-protein	96	GAGAGGGCGAGACAGTCACACT / CTCGTGGCTGTACTCTCCTCAGA
